# Are French local health contracts promising in addressing determinants of diet and physical activity?

**DOI:** 10.1093/eurpub/ckac129.590

**Published:** 2022-10-25

**Authors:** Y Le Bodo, D Diallo, H Hudebine, C Harpet, F Jabot, E Breton

**Affiliations:** 1 ARENES - UMR 6051, University Rennes, EHESP, CNRS, Rennes, France; 2 SHS, EHESP, Rennes, France; 3 LABERS, Université de Bretagne Occidentale, Brest, France; 4 Arènes - UMR 6051, RSMS - U 1309, University Rennes, EHESP, CNRS, Inserm, Rennes, France

## Abstract

**Background:**

There is a consensus on the need to transform the built, economic and socio-cultural environments to promote healthy eating (HE) and physical activity (PA). Yet, limited capacity and the biomedicalisation of public health are often blamed for steering investments towards individual risk factors. In France, local health contracts (LHCs) agreed between a regional health agency (RHA) and a local government could improve health promotion strategies. We examined how LHCs take into account the environmental determinants of HE and PA.

**Methods:**

Using the CLoterreS national census, we analysed a stratified random sample of 53 LHCs with a view to identify if actions target individual (knowledge, skills, etc.) or environmental (interpersonal, organisational, community or political) determinants. To this end, we developed an instrument drawing from different typologies of action and consensus documents to assess the integration of the socioecological approach in programmes. We ran a series of interviews with RHA staff (n = 39) and local actors (n = 23) to put into context our results.

**Results:**

Out of 53 LHCs, 42 included at least one action on HE or PA. For these topics, there was a higher proportion of actions targeting individuals (83% and 76% per contract, respectively) than environments (51% and 58%). For the latter, actions on interpersonal determinants (e.g. family) were the most common. However, we also found instances of actions on more distal ones (e.g. to improve nutrition standards in school canteens and walkability). Contextual factors such as local priorities, past experience with health promotion and the involvement of local actors in needs assessment and action planning may influence such orientations.

**Conclusions:**

LHCs constitute a promising avenue to address the environmental determinants of health-related behaviours. A key feature of this instrument is its capacity to develop intersectoral strategies. Further research will show if LHCs deliver on their action plan.

**Key messages:**

• Local health contracts facilitate the mobilisation of a broad diversity of NGOs and agencies.

• This makes it a promising device for addressing the environmental determinants of HE and PA, provided adequate resources are devoted to stakeholder engagement and local government capacity-building.

